# Two Cases and Review of the Literature: Primary Percutaneous Angiography and Antiplatelet Management in Patients with Immune Thrombocytopenic Purpura

**DOI:** 10.1155/2013/174659

**Published:** 2013-12-29

**Authors:** Estelle Torbey, Harout Yacoub, Donald McCord, James Lafferty

**Affiliations:** Staten Island University Hospital, Staten Island, NY 10305, USA

## Abstract

We report two cases of immune thrombocytopenic purpura (ITP) associated with acute coronary artery syndrome highlighting the interventions done in every case along with the medications used during intervention and as outpatient. The first case is that of a woman with ITP exacerbation while on dual antiplatelet therapy and the second case is that of a male presenting with non-ST elevation myocardial infarction (NSTEMI) while in a thrombocytopenic crisis. In both cases antiplatelet therapy was held and thrombopoietic therapy was initiated before resuming full anticoagulation and coronary intervention. Given the paucity of data on ITP and antiplatelets treatment in the setting of acute coronary syndrome, no strict recommendations can be proposed, but antiplatelets appear to be safe acutely and in the long term in this category of patients as long as few measures are undertaken to minimize the risks of bleeding and thrombosis.

## 1. Case 1

A 61-year-old smoker woman was admitted to the emergency department with retrosternal localized typical acute chest pain that started three days prior to presentation. Pain occurred at rest and was associated with shortness of breath. Upon arrival to the emergency room patient was still in pain, but vital signs were stable. The heart and lung examinations were remarkable for the presence of regular heart sounds with S3 gallop as well as bilateral fine basal crackles. Neither cutaneous nor mucosal petechia nor purpura was noted. The rest of the physical exam was normal. Her past medical history was significant for two uneventful pregnancies and chronic immune thrombocytopenic purpura (ITP) currently in remission. Ten years ago ITP was refractory with relapse during steroid tapering. Subsequently splenectomy was performed, establishing remission. Laboratory blood tests revealed hemoglobin of 15 mg/dL, platelet count of 322 × 10^9^/L, normal PT and PTT, troponin of 0.5 ng/mL, and CKMB 7.7 ng/mL. Inferior ST elevation was present on initial ECG with poor R wave progression anteriorly. She received in the emergency room nitroglycerin and morphine as well as 300 mg of clopidogrel and 325 mg of aspirin along with 5000 units of heparin bolus. Primary percutaneous coronary intervention was performed. The right common femoral artery was accessed with a 6 French sheath. Diagnostic angiography revealed acute 99% thrombotic occlusion of proximal LAD with an ejection fraction estimated at 25% on the left ventriculogram. The left circumflex and right coronary arteries were patent with right system dominance. A 6 French XBLAD 3.5 guiding catheter was used to intubate the left coronary system. Prior to stent placement, 4000 units of heparin and 0.25 mg/kg loading dose of abciximab were administered followed by a 0.125 mg/kg infusion of abciximab. An activated clotting time of 260 seconds was achieved. A 3.5∗20 mm tacrolimus eluted stent was deployed in the proximal segment of LAD with optimal angiographic result and TIMI flow 3 in the left anterior descending artery. The sheath introducer was removed after 6 hours with manual compression of the puncture site. Subsequently a small localized ecchymosis developed with spontaneous resolution in the following two days. The patient was discharged on aspirin 325 mg and clopidogrel 75 mg. Her platelet level upon discharge was 262 × 10^9^/L. Four years later she presented with a relapse of thrombocytopenia without active bleeding. All antiplatelets were held and intravenous steroids as well as intravenous immunoglobulins were administered. On the 3rd day platelets were 97 × 10^9^/L and clopidogrel was restarted with indication to restart aspirin after 2 weeks if platelet count remained stable.

Patient was readmitted within one month with mucosal hemorrhagic blisters and platelet count of 7 × 10^9^/L. A regimen of steroid and intravenous immunoglobulin pulse therapy was instituted with rise in platelet count to 90 × 10^9^/L on discharge. Clopidogrel was restarted. Rituximab was to be started at a later stage.

## 2. Case 2

A 55-year-old male smoker presented with typical anginal chest pain for three days. Upon arrival to the emergency room, pain was slightly improved and found to have NSTEMI with normal electrocardiogram and elevated troponins. The physical examination was unremarkable. The past medical history was significant for hypertension, dyslipidemia, and chronic ITP which responded to steroids and immunoglobulins in the past. Laboratory blood tests revealed a hemoglobin level of 15 mg/dL, platelet count of 42 × 10^9^/L, and normal PTT and PT. He was admitted to the coronary care unit and was started on simvastatin and nitroglycerin and clopidogrel was held. The patient was given one dose of IVIG and was started on prednisone. On the 7th day of hospitalization, the platelet count was 208 × 10^9^/L and the patient underwent cardiac catheterization through the right femoral artery. A drug eluted stent was placed in the obtuse marginal and a therapeutic ACT was reached after heparin administration. On the 8th day of hospitalization, the patient was discharged on aspirin 325 mg, clopidogrel 75 mg, and prednisone. Patient tolerated these medications well and remained in remission.

After six years, he presented again to the hospital with NSTEMI. The episode occurred one day after receiving dexamethasone and rituximab for ITP relapse. The physical exam was unremarkable and the platelet count was 23 × 10^9^/L. He received in the emergency room nitroglycerin and morphine as well as aspirin 81 mg and the patient was started on IV dexamethasone and IVIG. On the fifth day, the platelet count was 180 × 10^9^/L and the patient underwent cardiac catheterization through the femoral artery. Ostial triple vessel disease was treated with off pump bypass surgery with heparin to maintain an ACT > 300 through the procedure. The platelet count was 149 × 10^9^/L after receiving two days of dexamethasone and the patient was discharged on aspirin 325 mg orally daily. Both patients were followed up for two years without further complications.

## 3. Discussion

ITP is a disease characterized by IgG autoantibodies that bind to the platelets' surface leading to their phagocytosis in the reticuloendothelial system; this translates into thrombocytopenia and mucocutaneous bleeding. Myocardial infarction may occur when platelets are low, and when it occurs, careful balance between usual anticoagulation and antiplatelet therapy on one hand and efforts to raise platelet count on the other hand is needed. Acute coronary events have occurred in patients with ITP regardless of their platelet count which could range from normal to as low as 2 × 10^9^/L. Several incidences of myocardial infarction coincided with the administration of intravenous immunoglobulins (IVIG) or rituximab [1, 2]. The precise mechanism of thrombosis in ITP is unknown but has several explanations. First, it can be due to the presence of large immature prothrombotic platelets. These are mostly present during rituximab and IVIG therapy which induces an abrupt rise in the platelet count. Second, both platelets and endothelial cells can be targeted by anti-IIB/IIIA antibodies due to antigenic mimicry. Third, antiphospholipid antibodies are seen frequently in patients with ITP and have been reported to cause increased thrombosis [3]. Finally, patients with ITP are more prone to IVIG-related thrombotic arterial/venous complications including pulmonary embolism/deep venous thrombosis, myocardial infarction, and stroke [4, 5].

Acute coronary syndrome in patients with ITP represents a challenge in terms of therapeutic management. The literature was reviewed for cases of ITP who underwent a percutaneous coronary intervention (PCI). We found 22 admissions of patients with acute coronary syndrome (ACS) and ITP. The median age was 66 years. Among those, 47.36% were female and 52.89% were male. Twenty-five percent presented as NSTEMI, 45% as STEMI, while 25% had elective procedures performed. The mean platelet count on presentation was 66 ± 83 × 10^3^/mL. The PCI was performed in all cases except one. A femoral access was adopted in 80% of cases. Pretreatment with steroids, IVIG, and platelets was required in 52%, 27%, and 13% of cases, respectively. Danazol [6, 7] was used in two cases and romiplostim in two other cases [3, 8]. The platelet at the time of intervention was only known in 15 patients. The mean platelet count at which PCI was performed was 145 ± 87 × 10^9^/L. Major and minor bleeding occurred in 12% and 5% of cases, respectively. Heparin was used in 87% of cases (in 6 cases the anticoagulant agent was not mentioned). Hemostasis was achieved in 76.1% of cases by manual compression. No serious bleeding complications occurred as manual compression was used in most cases except for Park et al. [9] who used an occlusion device, the Angioseal. The patients were on single or dual antiplatelet therapy in 83% of cases. The longest follow-up period while on antiplatelets was 6 months.

The tendency to bleed due to the quantitative and qualitative deficiencies in the platelets is a concern during cardiac catheterization or coronary bypass while thrombolytics are contraindicated in patients with ITP [10]. The literature is noticeable for the lack of randomized controlled trials and for contradictory managements. A safe cutoff of platelet count above which invasive procedures can be performed has not been yet established. Russo et al. have suggested that when the platelet count is >50 × 10^9^/L intervention either percutaneously or surgically can be safely performed [11]. On the other hand, Russo et al. [11] had recommended the use of prednisolone prior to coronary artery bypass graft (CABG) in all patients with ITP regardless of the platelet count. The study included 30 patients undergoing CABG among which 9 received steroids and 19 received steroids and immunoglobulins. All 28 patients received platelet transfusion. At the time of the operation, twenty patients had the platelet count above 80 × 10^9^/L. The periprocedural treatment usually consists of methylprednisolone at a dose of 1 g/kg/d for 1–3 days alone or in addition to intravenous immunoglobulins. As for rituximab and thrombopoietin agonists, no clear data describes their role in the management of ITP pre- and post-PCI. We recommend that few measures are undertaken during the management of a coronary event in thrombocytopenic patients. First, coronary CT scan can be an alternative to angiography in low risk patients presenting with chest pain. It has been reported to be a safer primary method of diagnosis in patients with ITP when they present with chest pain but are at low risk for acute coronary syndrome [12]. Second, few points should be taken into consideration during angioplasty. Achieving hemostasis is one of the concerns during catheterization. Radial access results in lower bleeding rates and allows easier hemostatic compression, both of which are of major concern in patients with thrombocytopenia. As such radial access could be considered as the preferred site for patients with ITP. Nonetheless, femoral access was used in six cases followed by manual compression and only small ecchymosis that resolved spontaneously was reported [11, 13–15].

Aside from deciding on the access site, choosing the safest intravenous anticoagulant and antiplatelets (i.e., glycoprotein IIBIIIA) is another concern that arises during angioplasty. Unfractionated heparin (UFH) has been used in most cases without any significant complication [16, 17] while fondaparinux has been proposed as an alternative to UFH. Unlike UFH and LMWH, fondaparinux is a pure antithrombin III-dependent factor Xa inhibitor which has no effect on platelet function. This may potentially decrease the risk of serious bleeding in thrombocytopenic patients. [7] Neskovic et al. documented the successful use of half dose of UFH in addition to fondaparinux during PCI for ACS in an 80-year-old patient without bleeding complications.

The favorable outcome in the 80-year-old patient might suggest that this combination could be considered in patients with the highest periprocedural risk. Bivalirudin being a direct antithrombin is another alternative to heparin since it has been used in heparin-induced thrombocytopenia.

There is no clear data in the literature regarding glycoprotein IIBIIIA use in ITP patients presenting with ACS. Stouffer et al. described a case where the coadministration of clopidogrel and eptifibatide was not associated with significant bleeding [18]. Yagmur et al. have reported the safe administration of tirofiban that satisfactorily dissolved the thrombi and platelets remained constant at 54 × 10^9^/L without any bleeding event [12]. However, these two case reports do not eliminate the high probability of bleed while on GPIIbIIIa, and thus further prospective investigational studies are needed.

Third, the type of stent chosen will also have significant impact on the risk of bleeding in ITP patients. The type of stent will have an impact on the duration of dual antiplatelets administration as well as on the rate of restenosis. Among the cases found in the literature, only two cases deferred the deployment of a stent in the setting of ACS to avoid chronic dual antiplatelet therapy. Unfortunately, in both cases, reinfarction occurred which required urgent angioplasty [9, 11, 18]. Most expert opinions recommend bare metal stent placement as opposed to drug eluted stent to minimize the duration of dual antiplatelet therapy in case bleeding occurs [11, 19]. In our case, both patients received drug eluted stents because of the length of the lesions and their locations that predispose them to a high rate of restenosis.

Finally, the benefit of chronic administration of antiplatelets in case of ITP should be weighed against the prospective bleeding episodes that might occur during thrombocytopenia. In general individual case reports have shown that aspirin and clopidogrel are well tolerated even chronically when platelet count is above 30 × 10^9^/L [17]. Among those cases, 14 patients were discharged on aspirin [7, 8, 13–22], 15 patients received clopidogrel [6–8, 13–23], while two patients received abciximab, [8, 14] and two others received eptifibatide [8, 18]. One patient received ticlopidine [24] and three patients did not receive any antiplatelets [3, 25, 26]. While on antiplatelets, only one patient developed petechiae and nasal bleed that required clopidogrel to be stopped [18].

It seems that antiplatelet therapy was safe and well tolerated for long periods of time in all the reviewed cases and was only discontinued during a bleeding episode.

Acute or chronic atherosclerotic event in a patient with ITP is an intricate disease that involves the cardiologist as well as the hematologist. This multidisciplinary approach aims at managing a thrombotic event in the setting of a bleeding disorder. It is also required for the management of certain medication interactions. Thrombopoietin analogs have been known to be prothrombotic and an incident of stent thrombosis has been reported in a patient on romiplostim. The benefit of statin administration in patients using Danazol should be carefully balanced against the occurrence of rhabdomyolysis [27].

In conclusion, patients with history of ITP or patients with low platelet count presenting with ACS usually require a tailored medical and interventional management. Standardized therapy is recommended while patient is in remission. Patients presenting with low platelets (<30 × 10^9^/L) or with active bleed should be considered for pretreatment with steroids and IVIG while postponing, if possible, the treatment of the coronary syndrome; if treatment is required, the option remains to use bivalirudin. The administration of half the dose of unfractionated heparin with or without fondaparinux seems to be an alternative option for anticoagulation, but further studies are needed to prove the efficacy of such an antithrombotic approach. Coronary computerized tomography angiogram can be considered prior to PCI to rule out a thrombotic cause of acute coronary syndrome.

Finally, dual antiplatelet therapy appears to be tolerated in patients with ITP, but caution should be undertaken to withhold at least one of them during an ITP relapse.
